# Virtual reality in ENT medical education – pilot study of medical students’ perspective

**DOI:** 10.1017/S0022215126104708

**Published:** 2026-08

**Authors:** Anna Ewelina Kruczynska, Daniele Rimini, Prawin Samraj, Chaitanya Gadepalli

**Affiliations:** 1 School of Medicine, University of Dundee, Scotland, UK; 2 Medical Education, Raigmore Hospital, NHS Highlands, Inverness, Scotland, UK; 3 School of Medical Sciences, The University of Manchester, UK; 4 ENT Department, Salford Care Organisation, Northern Care Alliance NHS Foundation Trust, UK; 5 Department of Clinical Engineering, Salford Care Organisation, Northern Care Alliance NHS Foundation Trust, UK

**Keywords:** education, medical, larynx, models, anatomic, simulation training, virtual reality

## Abstract

**Objectives.:**

There is increasing demand for high-quality anatomy teaching for medical undergraduates. This study investigates medical students’ perspectives on using virtual reality to learn laryngeal anatomy.

**Methods.:**

Thirty-six Year 4 university teaching hospital medical students participated in a virtual reality laryngeal anatomy learning experience (Oculus Quest 2^®^ headsets). Students interacted with a VR model and completed a confidential feedback survey assessing their virtual reality exposure, learning environment, perceived usefulness and willingness to use virtual reality in future training.

**Results.:**

A total of 75 per cent of students had minimal prior virtual reality exposure. Despite this, 69.5 per cent agreed that virtual reality helped them learn the anatomy better than traditional lectures; 76.1 per cent believed virtual reality enhanced their learning; and 80 per cent expressed willingness to use virtual reality in their training. High satisfaction was reported regarding the learning environment, with 97.2 per cent feeling comfortable and engaged.

**Conclusions.:**

Virtual reality is a useful adjunct to laryngeal anatomy education, providing an interactive, engaging and resource-efficient adjunct to traditional methods.

## Introduction

Recently, there has been an increase in the number of medical students at universities across the United Kingdom,^1^^,^^2^ and a gradual phasing out of cadaveric dissections,^3^^,^^4^ resulting in the introduction of new challenges in teaching and learning anatomy. Medical education is evolving to meet these new challenges. Educational tools should be engaging and easy to use.

Alternative methods such as the use of prosections, simulation and online learning tools are gaining popularity.^5^^–^^7^ More recently, immersive technology such as virtual reality (VR) is being slowly adopted across the medical field. It involves the user putting on a VR headset to become immersed in an interactive virtual environment. The use of VR in anatomy teaching may lead to a broader availability of learning methods, reducing the need for specimens or cadavers, while maintaining the interactive aspect of learning.^8^^–^^12^ VR technology is currently used in our department for assessment of complex airways; a laryngeal anatomy teaching model was developed in a similar way in-house by the clinical engineering department at Salford Care Organisation using Unity® gaming software.

The VR experience was delivered using an Oculus Quest 2^®^ headset. It allowed students to interact with anatomical models of the larynx in various ways, such as moving around and rotating the models, grasping and scaling different parts of the larynx, looking inside the airway and viewing labels. Figure 1 shows the VR model in action. The larynx 3D model was obtained from an open-source website created by the University of Dundee.^13^

**Figure 1.**
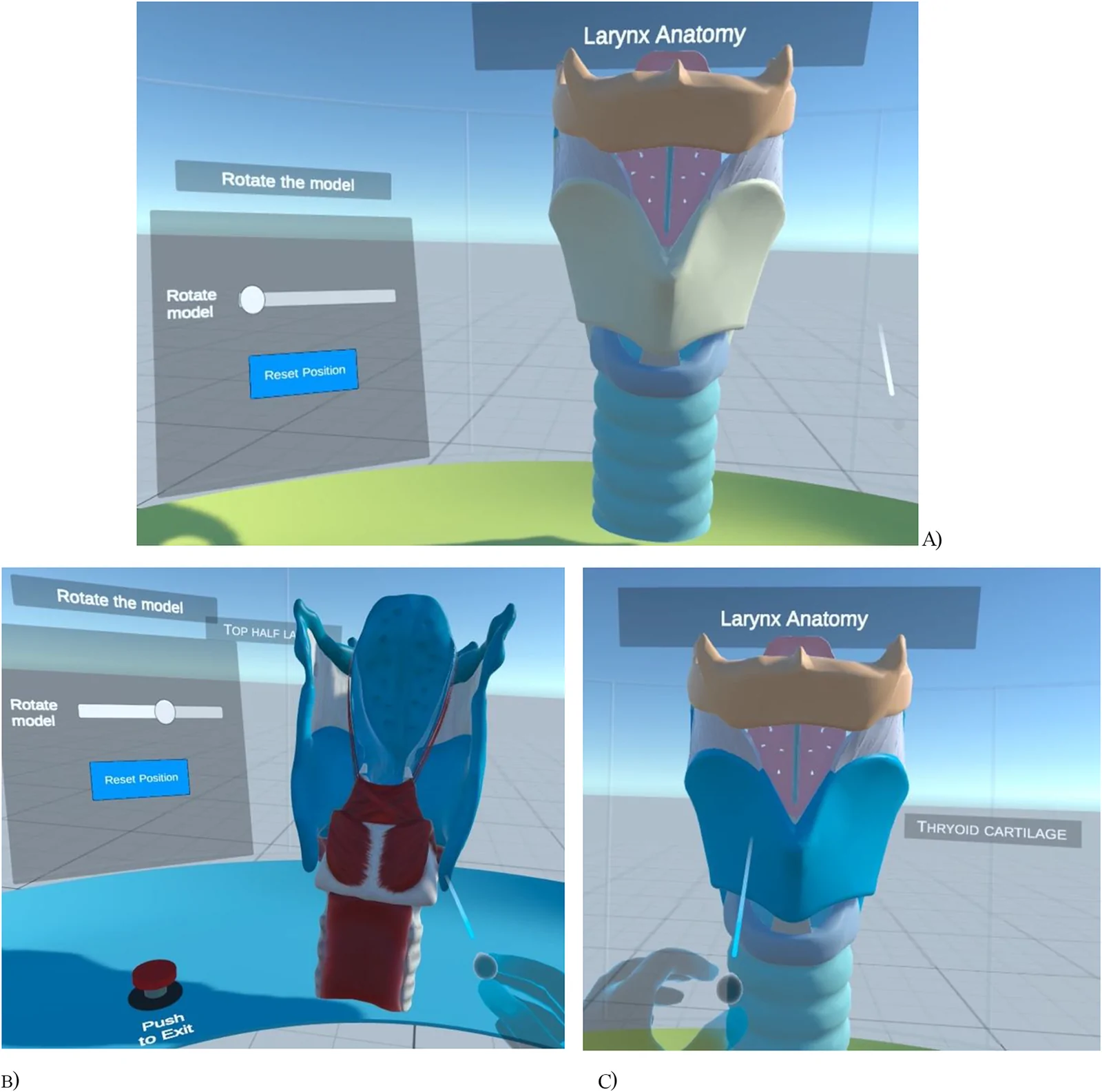
(A) The VR environment experienced by the participants. (B) How participants can rotate the model, reset the model to the original state by pressing the Reset Position button and exit the environment by pressing Push to Exit button. (C) Participant pointing to a structure and visualising a label.

Laryngeal anatomy was chosen because it is known to be especially difficult for medical students to learn using traditional methods.^14^ This study outlines the results of a survey conducted on Year 4 medical students who used VR to learn laryngeal anatomy.

## Materials and methods

Within the School of Medicine at a UK University, fourth-year medical students were given the opportunity to participate in a VR Laryngeal Anatomy learning experience as part of their ENT Clinical Placement. A confidential feedback survey was conducted, consisting of nine questions, as shown in Table 1. For the first eight questions, Likert five-point scale was used with the following responses strongly agree, agree, neutral, disagree and strongly disagree. The value of 4 or higher indicated agreement with the statement. The last question was a free text question: “What would have made this session better?” Cronbach’s Alpha was used to measure the internal consistency.

**Table 1.** Medical student survey questions and the corresponding results in percentage (%) for each point of five-point Likert scale Table 1 long description.

Number	Question	Agreement in percentage (%)				
		Strongly agree	Agree	Neutral	Disagree	Strongly disagree
1	I have had significant exposure to virtual reality (VR) in the past	5.6	11.1	8.3	22.2	52.8
2	VR helped me explore and learn topic better than a lecture would have	16.7	52.8	19.4	11.1	0
3	The learning environment was safe and supportive.	63.9	33.3	2.8	0	0
4	The session was engaging	48.6	48.6	0	2.8	0
5	I felt comfortable using the VR headset to navigate	52.8	44.5	2.7	0	0
6	I believe utilising VR would help me learn anatomy better.	61.1	25	8	5.9	0
7	I believe using VR would help me learn subject other than anatomy	36.1	41.7	16.7	5.5	0
8	I would use VR in my medical training if it was available to me.	60	20	14.3	5.7	0

## Results and analysis

The survey had 36 participants, yielding a 90 per cent response rate; 75 per cent did not have exposure to VR in the past. Table 1 illustrate the survey results. Cronbach’s Alpha score was 0.8, indicating there is high internal reliability of the questions asked.

The results indicate that 69.5 per cent of students agreed that VR has helped them explore and learn the topic better than a lecture would have, and 19.4 per cent of students answered neutrally. Additionally, 76.1 per cent of students agreed that VR would help them learn anatomy better, and 77.8 per cent agreed it would help with other subjects. If VR were available to students, 80 per cent of them agreed that they would use it in their learning. Students expressed a desire for longer VR sessions, more VR headsets and more VR activities.

## Discussion

The findings of this study show the potential of VR as an innovative tool in medical education, particularly for teaching complex anatomical structures such as the larynx. The high level of student response and positive feedback indicates that VR can effectively complement more traditional teaching methods. Most students reported that VR helped them understand the anatomy of the larynx better than traditional lectures, suggesting that immersive technology may have the potential to enhance spatial relations and acquisition of anatomical knowledge.

The study also revealed that students found the navigation of VR headsets to be comfortable and the learning environment to be safe and supportive. Student comfort and engagement are key factors in successful learning outcomes; therefore, the positive results of the survey support the benefits of using VR as a new teaching method. The high percentage of students willing to use VR in their medical training confirms the potential value of this technology.

However, the study also identified areas for improvement, such as the need for longer sessions and the provision of more VR headsets. VR headsets are expensive and creating a software program demands expertise from clinical engineering, which may not be available at every centre. However, with appropriate funding, these challenges may be overcome.

Assessing the economic feasibility for developing the application is essential to evaluate the sustainability and allocate resources appropriately. Although the final assembly time was approximately 5 hours, this underestimated the overall economic commitment required to acquire the necessary expertise. Consistent with established costing frameworks,^15^ we categorised development costs into five categories: personnel, equipment, software, facilities, other program inputs. Personnel costs included the shadow pricing of a software engineer with expertise in developing VR application, as well as a subject matter expert who provided instructions on the design and anatomical validation. Time allocated to address safety issues and data protection should be also included. Equipment costs consisted of the VR headset (about £300 per unit) and high-performance workstation. Software costs were minimal, as the primary development platform, Unity and Meta XR asset, are freely available for small-scale projects. Facilities costs reflected the use of physical office space and a dedicated VR testing area that must be kept free of obstacles to prevent user injuries. Finally, other program inputs that included costs for professional development to upskill the internal team and learner time to familiarise with the application and testing.

An additional cost component to consider is the anatomical 3D model. Although a freely available model was used in this work, it may be necessary to create patient-specific models or highly clinically accurate alternatives. Associated costs would include the technical workflow to execute the computed tomography/magnetic resonance imaging (CT/MRI) exam, image segmentation and the final validation by a clinician or radiologist. The final cost is affected by the level of details and information needed. Commercially available models range from approximately £100 for simple low-cost models to £400 for accurate multi-region models.

The use of VR as a teaching method in anatomy learning is still at its early stages of implementation. Further studies with larger number of participants are needed to explore its impact on learning outcomes and long-term knowledge retention. Future studies could investigate the use of VR across different anatomical regions, as well as its integration with other teaching methods. Addressing the cost-benefit analysis of using VR in an educational setting in greater detail, including its development cost and learning outcomes, would also be beneficial.

- Medical education is evolving leading to new challenges
- Immersive technologies such as virtual reality (VR) compliment interactive education
- Majority find the VR technology in medical education useful and improves understanding
- Collaboration with clinical engineering and funding are key to implementation of this technology

## Conclusion

VR has the potential to improve anatomy education by providing an interactive, engaging and efficient adjunct to traditional methods. With the constant evolution of medical education, incorporating innovative technologies like VR will be useful in meeting the challenges of teaching complex anatomical concepts to students and trainees.

## Table 1 Long description

The table reports medical students’ responses to eight statements about prior virtual reality experience and perceptions of VR-based learning, using a five-point agreement scale with percentages. Prior exposure was low: most strongly disagreed they had significant past VR exposure, and only a small minority agreed. Despite that, students viewed VR positively for learning: most agreed or strongly agreed that VR helped them learn better than a lecture, with very few disagreeing. Perceptions of the learning setting were especially strong, with nearly all students agreeing it was safe and supportive and almost none expressing disagreement. Engagement and usability were also rated highly, with most students agreeing the session was engaging and that they felt comfortable navigating with the headset. Students largely believed VR would help them learn anatomy, and many also thought it would help with subjects beyond anatomy, though agreement was somewhat lower and neutral responses higher for non-anatomy topics. Intent to use VR in medical training if available was high, with most agreeing or strongly agreeing and minimal disagreement. Percentages reflect self-reported attitudes from a single survey and do not by themselves indicate learning outcomes or generalize beyond the surveyed group.

Navigate back to Table 1.
